# Workers’ Memorial Day — April 28, 2013

**Published:** 2013-04-26

Workers’ Memorial Day recognizes workers who died or suffered from exposures to hazards at work. In 2011, a total of 4,069 U.S. workers died from work-related injuries (1). Most fatalities from work-related illness are not captured by national surveillance systems, but an estimate for 2007 was 53,445 deaths (2). Several national surveillance systems report new cases of nonfatal work-related injuries and illnesses, although no system captures all cases. In 2011, nearly 3 million injuries and illnesses to private industry workers and 821,000 to state and local government workers were reported by employers (3). In the same year, an estimated 2.9 million work-related injuries were treated in emergency departments, resulting in 150,000 hospitalizations (CDC, unpublished data, 2013).

Based on methods that focus on medical costs and productivity losses, the societal cost of work-related fatalities, injuries, and illnesses was estimated at $250 billion in 2007 (2). Methods that include consideration of pain and suffering would result in a higher estimated societal cost (4). CDC is working to better describe the burden of fatalities, injuries, and illnesses suffered by workers; additional information is available at http://www.cdc.gov/niosh/programs/econ/risks.html.
