# Erratum

**Published:** 2013-05-10

## Vol. 62, No. 16

An error occurred on page 301, in the second sentence of the box, “Workers’ Memorial Day — April 28, 2013.” That sentence should read, “In 2011, a total of **4,609** U.S. workers died from work-related injuries (1).” However, this number was based on preliminary data from the Census of Fatal Occupational Injuries, which have since been revised and finalized. The final count of fatal work injuries in the United States in 2011 is **4,693**. Additional information is available at http://www.bls.gov/iif/oshwc/cfoi/cfoi_revised11.pdf.

